# Shear Wave Elastography for Parotid Glands: Quantitative Analysis of Shear Elastic Modulus in Relation to Age, Gender, and Internal Architecture in Patients with Oral Cancer

**DOI:** 10.3390/jimaging11050145

**Published:** 2025-05-04

**Authors:** Yuka Tanabe, Ai Shirai, Ichiro Ogura

**Affiliations:** 1 Quantitative Diagnostic Imaging, Field of Oral and Maxillofacial Imaging and Histopathological Diagnostics, Course of Applied Science, The Nippon Dental University Graduate School of Life Dentistry at Niigata, 1-8 Hamaura-cho, Chuo-ku, Niigata 951-8580, Japan; y.tanabe@ngt.ndu.ac.jp (Y.T.); ai@ngt.ndu.ac.jp (A.S.); 2 Department of Oral and Maxillofacial Radiology, The Nippon Dental University School of Life Dentistry at Niigata, 1-8 Hamaura-cho, Chuo-ku, Niigata 951-8580, Japan

**Keywords:** elasticity imaging technique, ultrasonography, parotid gland

## Abstract

Background: Recently, shear wave elastography (SWE) has been recognized as an effective tool for evaluating Sjögren’s syndrome (SS) patients. The purpose of this study was to assess the parotid glands with SWE, especially for quantitative analysis of shear elastic modulus in relation to age, gender, and internal architecture in patients with oral cancer to collect control data for SS. Methods: In total, 124 parotid glands of 62 patients with oral cancer were evaluated with SWE. The parotid glands were examined for the internal architecture (homogeneous or heterogeneous) on B-mode. The SWE allowed the operator to place regions of interest (ROIs) for parotid glands, and displayed automatically shear elastic modulus data (kPa) for each ROI. Gender and internal architecture were compared with the shear elastic modulus of the parotid glands by Mann–Whitney U-test. The comparison of age and shear elastic modulus was assessed using Spearman’s correlation coefficient. *p* < 0.05 was considered statistically significant. Results: The shear elastic modulus of the parotid glands was not significantly different for according to gender (males, 7.70 ± 2.22 kPa and females, 7.67 ± 2.41 kPa, *p* = 0.973) or internal architecture (homogeneous: 7.69 ± 2.25 kPa and heterogeneous: 7.72 ± 2.74 kPa, *p* = 0.981). Furthermore, the shear elastic modulus was not correlated with age (*n* = 124, R = −0.133, *p* = 0.139). Conclusion: Our study showed the control data of the shear elastic modulus of the parotid glands for SS. SWE is useful for the quantitative evaluation of the parotid glands.

## 1. Introduction

Ultrasonography is useful as a diagnostic modality in oral and maxillofacial lesions, and power Doppler sonography is an effective modality in the differential diagnosis of buccal space tumors [1]. Recently, a few reports involving intraoral ultrasonography have been published on tongue carcinoma [2], palatal tumors [3], gingival carcinoma [4], and palatal lymphoma [5]. Furthermore, strain elastography with intraoral ultrasonography is useful for differentiating tongue carcinoma [2] and palatal tumors [3,5].

Recently, shear wave elastography (SWE) has been recognized an effective tool because of the quantitative diagnosis for head and neck diseases, such as squamous cell carcinoma (SCC) [6], cervical lymph nodes [7], masseter muscle [8], and buccal cyst [9]. SWE is a recently developed technique that uses push pulses to put stress on tissues and an ultrafast ultrasound imaging technique to detect the induced shear waves, and shear elastic modulus data have been determined in different tissues [6].

Sjögren’s syndrome (SS) is a chronic autoimmune disease characterized by dry eyes and mouth resulting from lacrimal and salivary gland dysfunction [10]. Scintigraphy is an effective modality for evaluating salivary gland dysfunction [11]. Magnetic resonance (MR) sialography is a functional method of study for salivary glands in patients with SS [12]. Furthermore, MR imaging and salivary gland scintigraphy are effective methods for assessing patients with SS during follow up [13]. In recent years, salivary gland SPECT/CT has been recognized as an effective tool in assessing patients with salivary gland dysfunction [14,15,16]. However, the problem with current diagnostic tools used for salivary gland scintigraphy, MR sialography, and SPECT/CT, is that they are not simple or easy to use, not found in any hospitals, and costly.

Ultrasonography of the salivary glands is useful in evaluating SS [17,18,19,20,21]. Ultrasonography of the salivary glands is effective in visualizing glandular structural changes in patients suspected of having SS [17]. Elastography with ultrasonography is effective specifically in the diagnosis of SS [18]. Ultrasonography of the salivary glands is a noninvasive technique with good sensitivity and specificity in diagnostic aid for SS [19].

SWE should be essential in the quantitative diagnosis of the parotid glands in SS. However, a few reports have been published on SWE in the investigation of salivary glands in SS [22,23]. Collecting data on salivary glands with SWE is necessary for medical treatment of SS. Unfortunately, no reports have been published on the evaluation of parotid glands using SWE, such as age, gender, and internal architecture. SWE is useful for the quantitative diagnosis for oral cancer lymph node metastases [7], and many patients with oral cancer undergo SWE in the head and neck. Therefore, we investigated the parotid glands with SWE, especially quantitative analysis of shear elastic modulus in relation to age, gender, and internal architecture in patients with oral cancer to establish control data for the parotid glands for SS.

## 2. Materials and Methods

All patients provided written informed consent, and this prospective study was approved by the ethics committee of The Nippon Dental University School of Life Dentistry at Niigata (approved no. ECNG-R-400). In total, 124 parotid glands of 62 patients (41 men and 21 women; mean age 69.3 years (age 30–89 years)) after only primary surgery for oral SCC (without chemotherapy and/or radiotherapy against oral cancer) were evaluated using an ultrasonographic unit with a 14 MHz linear transducer (Aplio 300, Canon Medical Systems, Otawara, Japan) at The Nippon Dental University Niigata Hospital from May 2024 to January 2025, following the hospital’s protocol [23]. The diagnoses of oral cancer were obtained by surgeons in all cases.

**Figure 1:**
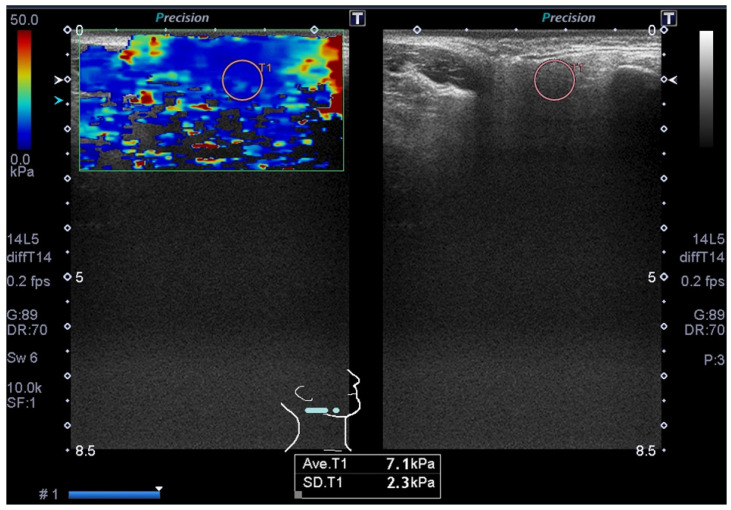
Ultrasonography of right parotid gland in 68-year-old male with tongue carcinoma. Ultrasonography indicates homogeneity in internal architecture of parotid gland on B-mode. Shear wave elastography indicates that shear elastic modulus of parotid gland is 7.1 ± 2.3 kPa.

**Figure 2:**
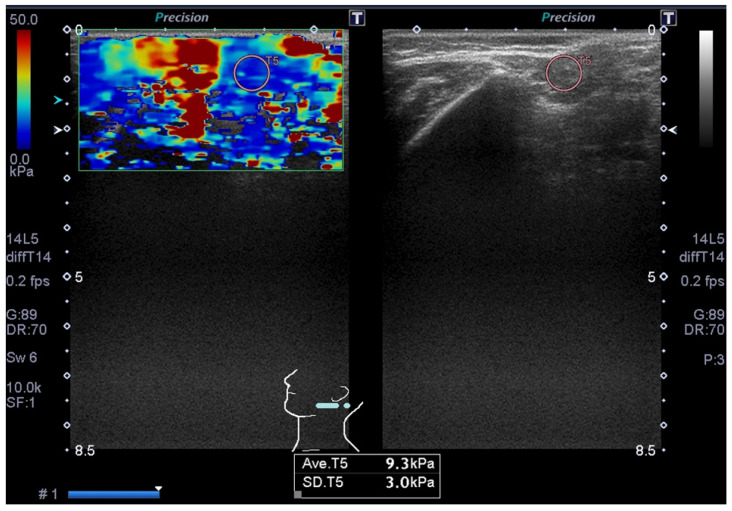
Ultrasonography of left parotid gland in a 75-year-old female with tongue carcinoma. Ultrasonography indicates heterogeneity in internal architecture of parotid gland on B-mode. Shear wave elastography indicates that shear elastic modulus of parotid gland is 9.3 ± 3.0 kPa.

Two oral radiologists reviewed all of the images, and any discrepancies were resolved by consensus. In each patient, the right and left parotid glands were evaluated with ultrasonography. The parotid glands were assessed via the internal architecture (homogeneous or heterogeneous) on B-mode. Furthermore, in each patient, the right and left parotid glands were evaluated with SWE, following our hospital’s protocol [23]. The examiner used regions of interests (ROIs) to calculate the shear elastic modulus data (kPa) of the right and left parotid glands within the elastography window, and automatically displayed shear elastic modulus data for each ROI (Figure 1 and Figure 2).

The gender and internal architecture were compared with the shear elastic modulus of the parotid glands using Mann–Whitney U-tests and Pearson’s chi-square tests because non-parametric data were being used. The comparison of age and shear elastic modulus was performed using Spearman’s correlation coefficient because non-parametric data were being used. The data were evaluated using a statistical package (IBM SPSS Statistics version 26, IBM Japan, Tokyo, Japan). *p* < 0.05 was considered statistically significant.

## 3. Results

The shear elastic modulus of the parotid glands were not significantly different for gender (males: 7.70 ± 2.22 kPa and females: 7.67 ± 2.41 kPa, *p* = 0.973) or internal architecture (homogeneous: 7.69 ± 2.25 kPa and heterogeneous: 7.72 ± 2.74 kPa, *p* = 0.981) (Table 1).

Regarding internal architecture, homogeneity was 95.1% in males and 88.1% in females, and heterogeneity was 4.9% in males and 11.9% in females, respectively (*p* = 0.153). Furthermore, the shear elastic modulus of the parotid glands was not significantly different for homogeneity (males: 7.72 ± 2.20 kPa and females: 7.61 ± 2.39 kPa, *p* = 0.762) or heterogeneity (males: 7.15 ± 2.90 kPa and females: 8.18 ± 2.85 kPa, *p* = 0.730) (Table 2).

Figure 1 shows the ultrasonography of a right parotid gland in a 68-year-old male with tongue carcinoma. The ultrasonography indicates homogeneity in the internal architecture of the parotid gland. SWE indicates that the shear elastic modulus of the parotid gland is 7.1 kPa. Furthermore, Figure 2 shows the ultrasonography of a left parotid gland in a 75-year-old female with tongue carcinoma. The ultrasonography indicates heterogeneity in the internal architecture of the parotid gland. SWE indicates that the shear elastic modulus of the parotid gland is 9.3 kPa.

Furthermore, the shear elastic modulus was not correlated with age (n = 124, R = −0.133, *p* = 0.139).

## 4. Discussion

We investigated the parotid glands with shear wave elastography, especially quantitative analysis of the shear elastic modulus in relation to age, gender, and internal architecture in patients with oral cancer, and concluded that the shear elastic modulus of the parotid glands was not significantly different according to gender or internal architecture. Furthermore, the shear elastic modulus was not correlated with age.

Cindil et al. [18] indicated that strain elastography could play a roll in the differential diagnosis of SS. Bukhari et al. [19] indicated that salivary gland ultrasonography is a noninvasive imaging modality that can be used as a diagnostic aid for SS. Kise et al. [20] suggest that the evaluation of salivary glands using ultrasonographic elastography is an effective tool for the diagnosis of SS. Dai et al. [21] indicated that ultrasonographic elastography is a useful tool in the diagnosis of SS. However, a few reports have been published on SWE in the evaluation of salivary glands, such as the parotid glands. In this study, we showed that SWE is an effective tool for the quantitative evaluation of the parotid glands.

Regarding the internal architecture of the parotid glands on ultrasonographic findings, Salaffi et al. [17] indicated that 66/77 (85.7%) patients with SS had abnormal ultrasonographic findings. Shirai et al. [23] showed that 83.3% of patients with SS and 3.4% of patients with oral cancer as the control group showed heterogeneity in the internal architecture of the parotid glands on ultrasonographic findings. In this study, 9/124 (7.3%) parotid glands in patients with oral cancer showed heterogeneity in internal architecture. We suggest that salivary gland ultrasonography is an effective technique in visualizing glandular structural changes in patients suspected of having SS, and it could represent a good option as a first-choice imaging method for SS.

Arslan et al. [22] showed that the shear elastic modulus of the parotid glands for SS (right: 32.2 ± 16.1 kPa and left: 37.9 ± 19.1 kPa) was higher than those of controls (right: 14.4 ± 5.7 kPa and left: 13.2 ± 4.1 kPa). Shirai et al. [23] indicated that the shear elastic modulus of the parotid glands for SS (11.9 ± 4.3 kPa) was higher than those of oral cancer as controls (8.3 ± 3.2 kPa), and SS and oral cancer were not significantly different according to age. In this study, the shear elastic modulus of the parotid glands in patients with oral cancer were not significantly different according to gender (males: 7.70 ± 2.22 kPa and females: 7.67 ± 2.41 kPa) or internal architecture (homogeneous: 7.69 ± 2.25 kPa and heterogeneous: 7.72 ± 2.74 kPa); furthermore, the shear elastic modulus was not correlated with age. Therefore, none of the comparisons for the shear elastic modulus of the parotid glands reached statistical significance. This study assessed the shear elastic modulus of the parotid glands in oral cancer for the evaluation for control data for SS. We emphasize the clinical importance of non-significant trends is the establishment of control data for the shear elastic modulus of the parotid glands for SS. Further research on the parotid glands in patients with SS using SWE is necessary for clinical importance.

Regarding SWE, Ogura et al. [6] indicated that the mean shear elastic modulus of the sublingual gland, geniohyoid muscle, and anterior belly of the digastric muscle was 9.4 kPa, 19.2 kPa, and 15.3 kPa, respectively. Furthermore, the maximum shear elastic modulus of SCCs (109.6 kPa) was higher than that of benign lesions (46.4 kPa). Sasaki et al. [7] showed that that of the malignant cervical lymph nodes (105.9 kPa) were higher than that of benign ones (11.9 kPa). Minami et al. [8] indicated that that of masseter muscles at contraction (147.6 kPa) were significantly higher than that during rest (27.2 kPa). In this study, the parotid glands displaying homogeneity were found in males, 7.72 kPa, and females, 7.61 kPa, and those displaying heterogeneity were males, 7.15 kPa, and females, 8.18 kPa. We concluded that the data could be used for reference in future studies and clinical settings for salivary gland lesions.

The assessment of salivary glands using ultrasonography is a useful diagnostic technique for SS [17,18,19,20,21]. Salivary gland ultrasonography is an effective modality for visualizing glandular structural changes in patients suspected of having SS [17]. Strain elastography is an effective modality for the differential diagnosis of SS [18]. Furthermore, salivary gland ultrasonography is a noninvasive imaging technique with good sensitivity and specificity for SS [19]. We showed that SWE is useful for the quantitative evaluation of the parotid glands, and consider SWE to be an effective modality for the management of patients with SS.

While SWE has been explored in SS and other salivary gland conditions, its use in cancer patients is less common. We studied the shear elastic modulus of the parotid glands in oral cancer for the of establishment of control data for SS. Furthermore, Ogura et al. [24] indicated that the structural variations in parotid glands induced by radiotherapy were density changes. We suggest that the SWE of parotid glands in patients with oral cancer is useful for potential radiation therapy planning or post-treatment monitoring roles.

The study lacks histopathologic confirmation or functional salivary gland assessment, which would be needed to validate the SWE measurements. Without comparing SWE with clinical markers of gland function or histologic fibrosis, the utility of the stiffness values remains speculative. Therefore, further research on those is necessary and clinically important.

There are some limitations to this study. The number of cases we used was relatively small (124 parotid glands of 62 patients with oral cancer) for a study of shear wave elastography for parotid glands. But Salaffi et al. [17] conducted an ultrasonography study of salivary glands in 77 patients with SS. Furthermore, Cindil et al. [18], Arslan et al. [22], and Shirai [23] studied 58, 53, and 6 patients with SS using ultrasound elastography, respectively. It was clear that other studies have also used a smaller number of samples for research. Furthermore, those patients were not asked about the point of comparison with pathological or clinical findings on parotid glands. Further research on parotid glands using SWE is necessary to validate these results.

## 5. Conclusions

Our study highlights the shear wave elastography for parotid glands, especially quantitative analysis of the shear elastic modulus in relation to age, gender, and internal architecture in patients with oral cancer as the control data for SS. By focusing on patients with oral cancer, we aimed to evaluate parotid glands with SWE. Our findings demonstrated that the shear elastic modulus of the parotid glands were not significantly different according to gender or internal architecture. Furthermore, the shear elastic modulus was not correlated with age. Although the results are encouraging, further validation on larger multicenter datasets is needed. Future research using SWE should aim to evaluate the salivary glands in patients with SS.

This study employed control data for the shear elastic modulus of the parotid glands in SS patients. SWE is useful for the quantitative evaluation of the parotid glands.

## Figures and Tables

**Table 1:** Shear wave elastography of parotid glands in patients with oral cancer.

Parotid Glands	Shear Elastic Modulus (kPa)	*p*-Value
(n = 124)	Mean ± Standard Deviation (Range)	
Gender		0.973
Males (n = 82)	7.70 ± 2.22 (3.7–13.2)	
Females (n = 42)	7.67 ± 2.41 (3.8–14.5)	
Internal architecture		0.981
Homogeneous (n = 115)	7.69 ± 2.25 (3.7–14.5)	
Heterogeneous (n = 9)	7.72 ± 2.74 (3.8–11.4)	

**Table 2:** Shear wave elastography of parotid glands in internal architecture for gender.

Parotid Glands (n = 124)	Shear Elastic Modulus (kPa)		*p*-Value
	Mean ± Standard Deviation (Range)		
	Males (n = 82)	Females (n = 42)	
Internal architecture			0.153
Homogeneous (n = 115)	n = 78 (95.1%)	n = 37 (88.1%)	
	7.72 ± 2.20 (3.7–13.2)	7.61 ± 2.39 (4.0–14.5)	0.762
Heterogeneous (n = 9)	n = 4 (4.9%)	n = 5 (11.9%)	
	7.15 ± 2.90 (4.9–11.4)	8.18 ± 2.85 (3.8–10.7)	0.730

## Data Availability

The data presented in this study are available on request from the corresponding author.

## References

[1] Ogura I., Kaneda T., Sasaki Y., Sekiya K., Tokunaga S. (2013). Characteristic power Doppler sonographic images of tumorous and non-tumorous buccal space lesions. Dentomaxillofac. Radiol..

[2] Ogura I., Sasaki Y., Sue M., Oda T. (2018). Strain elastography of tongue carcinoma using intraoral ultrasonography: A preliminary study to characterize normal tissues and lesions. Imaging Sci. Dent..

[3] Ogura I., Toshima H., Akashiba T., Ono J., Okada Y. (2020). Strain elastography of palatal tumors in conjunction with intraoral ultrasonography, computed tomography, and magnetic resonance imaging: 2 cases reports. Imaging Sci. Dent..

[4] Minami Y., Okada Y., Ogura I. (2023). Intraoral ultrasonography for gingival squamous cell carcinoma: Tumor thickness and bone invasion. Oral Sci. Int..

[5] Minami Y., Ogawa R., Kanri Y., Tezuka Y., Okada Y., Ogura I. (2023). Characteristic multimodal imaging of palatal follicular lymphoma: A case report on effectiveness of CT, diffusion-weighted MR imaging and intraoral ultrasonography. Oral Radiol..

[6] Ogura I., Nakahara K., Sasaki Y., Sue M., Oda T. (2018). Usefulness of shear wave elastography in the diagnosis of oral and maxillofacial diseases. Imaging Sci. Dent..

[7] Sasaki Y., Ogura I. (2019). Shear wave elastography in differentiating between benign and malignant cervical lymph nodes in patients with oral carcinoma. Dentomaxillofac. Radiol..

[8] Minami Y., Ogura I. (2022). Quantitative analysis of masseter muscle hardness with shear-wave elastography: Preliminary study on comparison between during rest and contraction in young adults. J. Oral Maxillofac. Radiol..

[9] Tezuka Y., Oneyama T., Kanri Y., Toya S., Okada Y., Ogura I. (2024). A case of odontogenic keratocyst in the buccal space: Characterization by multimodality imaging including computed tomography, diffusion-weighted magnetic resonance imaging, and ultrasonography. Oral Radiol..

[10] Negrini S., Emmi G., Greco M., Borro M., Sardanelli F., Murdaca G., Indiveri F., Puppo F. (2022). Sjögren’s syndrome: A systemic autoimmune disease. Clin. Exp. Med..

[11] Ogura I., Hayama K., Sue M., Oda T., Sasaki Y. (2017). Submandibular sialolithiasis with CT and scintigraphy: CT values and salivary gland excretion in the submandibular glands. Imaging Sci. Dent..

[12] Cho A., Lee Y.R., Jeon Y.T., Chang S.-H., Park Y.M., Ahn S.J., Lim J.-Y. (2023). Correlations of MR sialographic gradings with the clinical measures of Sjögren’s syndrome. Laryngoscope.

[13] Ogura I., Sasaki Y., Oda T., Sue M., Hayama K. (2018). Magnetic resonance sialography and salivary gland scintigraphy of parotid glands in Sjögren’s syndrome. Chin. J. Dent. Res..

[14] Ninomiya K., Toya S., Ogura I. (2020). Single-photon emission computed tomography/computed tomography for evaluation of salivary gland dysfunction: Preliminary study on diagnostic ability of maximum standardized uptake value. Oral Radiol..

[15] Shirai A., Ogura I. (2025). Maximum standardized uptake value for parotid and submandibular glands in patients with Sjögren’s syndrome and submandibular sialolithiasis using salivary gland SPECT/CT. Odontology.

[16] Tanabe Y., Ogura I. (2025). Submandibular sialolithiasis with CT and SPECT/CT: CT values, standardized uptake values, and salivary gland excretion in the parotid and submandibular glands. Dentomaxillofac. Radiol..

[17] Salaffi F., Carotti M., Iagnocco A., Luccioli F., Ramonda R., Sabatini E., De Nicola M., Maggi M., Priori R., Valesini G. (2008). Ultrasonography of salivary glands in primary Sjögren’s syndrome: A comparison with contrast sialography and scintigraphy. Rheumatology.

[18] Cindil E., Oktar S.O., Akkan K., Sendur H.N., Mercan R., Tufan A., Ozturk A. (2018). Ultrasound elastography in assessment of salivary glands involvement in primary Sjögren’s syndrome. Clin. Imaging.

[19] Bukhari A.F., Farag A., Papas A., Ganguly R., Campos H., Ramesh A. (2021). Salivary glands ultrasonography as a diagnostic aid in Sjögren’s syndrome: A prospective pilot investigation. Oral Surg. Oral Med. Oral Pathol. Oral Radiol..

[20] Kise Y., Møystad A., Kuwada C., Ariji E., Bjørnland T. (2024). Does ultrasound elastography have a role as a diagnostic method for Sjögren’s syndrome in the salivary glands? A systematic review. Oral Radiol..

[21] Dai X., Sui X., Chen S., Zhao B., Liu Z., Wang X. (2024). The diagnostic performance of salivary gland ultrasound elastography in Sjögren’s syndrome and sicca symptoms: A systematic review and meta-analysis. Eur. Radiol..

[22] Arslan S., Durmaz M.S., Erdogan H., Esmen S.E., Turgut B., Iyisoy S. (2020). Two-dimensional shear wave elastography in the assessment of salivary gland involvement in primary Sjögren’s syndrome. J. Ultrasound Med..

[23] Shirai A., Ogawa R., Tezuka Y., Nakatani Y., Toya S., Ogura I. (2025). A prospective pilot study on shear wave elastography: Evaluation of parotid glands in Sjögren’s syndrome and comparison with oral cancer cases. Oral Sci. Int..

[24] Ogura I., Sasaki Y., Oda T., Sue M., Yamaguchi H., Kameta A., Hayama K., Tsuchimochi M. (2017). Structural variations in parotid glands induced by radiation therapy in patients with oral carcinoma observed on contrast-enhanced computed tomography. Pol. J. Radiol..

